# Laparoscopic versus robotic-assisted, left-sided colectomies: intra- and postoperative outcomes of 683 patients

**DOI:** 10.1007/s00464-021-09003-x

**Published:** 2022-01-13

**Authors:** Jörn-Markus Gass, Diana Daume, Romano Schneider, Daniel Steinemann, Francesco Mongelli, Andreas Scheiwiller, Lana Fourie, Beatrice Kern, Markus von Flüe, Jürg Metzger, Fiorenzo Angehrn, Martin Bolli

**Affiliations:** 1 Department of General Surgery, Cantonal Hospital of Lucerne, Spitalstrasse, 6000 Lucerne, Switzerland; 2 Department of Health Sciences and Medicine, University of Lucerne, Frohburgstrasse 3, 6002 Lucerne, Switzerland; 3 Clarunis, Department of Visceral Surgery, University Centre for Gastrointestinal and Liver Diseases, St. Clara Hospital and University Hospital Basel, Kleinriehenstrasse 30, 4058 Basel, Switzerland; 4 Department of Surgery, Regional Hospital of Lugano, Via Tesserete 46, 6900 Lugano, Switzerland

**Keywords:** Laparoscopic surgery, Robotic-assisted surgery, Left-sided colectomies

## Abstract

**Background:**

Robotic-assisted colorectal surgery has gained more and more popularity over the last years. It seems to be advantageous to laparoscopic surgery in selected situations, especially in confined regions like a narrow male pelvis in rectal surgery. Whether robotic-assisted, left-sided colectomies can serve as safe training operations for less frequent, low anterior resections for rectal cancer is still under debate. Therefore, the aim of this study was to evaluate intra- and postoperative results of robotic-assisted laparoscopy (RAL) compared to laparoscopic (LSC) surgery in left-sided colectomies.

**Methods:**

Between June 2015 and December 2019, 683 patients undergoing minimally invasive left-sided colectomies in two Swiss, high-volume colorectal centers were included. Intra- and postoperative outcome parameters were collected and analyzed.

**Results:**

A total of 179 patients undergoing RAL and 504 patients undergoing LSC were analyzed. Baseline characteristics showed similar results. Intraoperative complications occurred in 0.6% of RAL and 2.0% of LSC patients (*p* = 0.193). Differences in postoperative complications graded Dindo ≥ 3 were not statistically significant (RAL 3.9% vs. LSC 6.3%, *p* = 0.227). Occurrence of anastomotic leakages showed no statistically significant difference [RAL *n* = 2 (1.1%), LSC *n* = 8 (1.6%), *p* = 0.653]. Length of hospital stay was similar in both groups. Conversions to open surgery were significantly higher in the LSC group (6.2% vs.1.7%, *p* = 0.018), while stoma formation was similar in both groups [RAL *n* = 1 (0.6%), LSC *n* = 5 (1.0%), *p* = 0.594]. Operative time was longer in the RAL group (300 vs. 210.0 min, *p* < 0.001).

**Conclusion:**

Robotic-assisted, left-sided colectomies are safe and feasible compared to laparoscopic resections. Intra- and postoperative complications are similar in both groups. Most notably, the rate of anastomotic leakages is similar. Compared to laparoscopic resections, the analyzed robotic-assisted resections have longer operative times but less conversion rates. Further prospective studies are needed to confirm the safety of robotic-assisted, left-sided colectomies as training procedures for low anterior resections.

Over the last few decades, minimally invasive surgery has gained rising popularity. With the adoption of laparoscopic techniques, several advantages concerning length of hospital stay, level of postoperative pain, blood loss, and reduction of postoperative complications as well as faster return to normal activities could be demonstrated. Now even complex procedures can be performed with at least equal and non-inferior results compared to the open technique [1–4]. These advantages have also been proven for oncological resections, demonstrating non-inferiority in regards to oncological outcomes [5, 6]. The implementation of robotic-assisted systems took minimally invasive surgery to the next level. The Endowrist™ function, with its improvement in comfort and maneuverability, filtration of tremor and motion by the computer system, high-definition 3-dimensional binocular vision with the option of magnification, fluorescence, and finally, a stable platform with a surgeon operated camera, can even be regarded as a revolution and new dimension in surgery [7–9].

The main advantages of robotic-assisted surgery have been described in procedures with confined spaces like in rectal and esophageal surgery. Until now, the lack of evidence and the higher costs of the new technique have limited a more extensive adoption of the robotic-assisted systems [10, 11]. Comparative studies between robotic-assisted and laparoscopic techniques were able to show that robotic-assisted abdominal surgery is safe, feasible, and at least equivalent in regards to short-term outcomes [12, 13]. Clinical results are encouraging: conversion rates to open surgery, length of hospital stay, return to normal activities and work as well as recovery time are comparable [14, 15]. On the other hand, skeptics claim higher costs, longer operative time due to docking and redocking and the rather long learning curve [16, 17]. Concerning colorectal surgery, the learning curve is estimated to comprise 35 procedures. In general, low anterior resections for rectal cancer are performed less frequently than colonic resections. Left-sided colectomies are performed routinely for either benign diseases like diverticulitis and symptomatic diverticulosis or for colon cancer. Essential parts of the procedure are part of low anterior resections as well. Therefore, the question arises, if left-sided colectomies can be performed safely as robotic-assisted training procedures to prepare for low anterior rectal resections.

The aim of the present study was to analyze intra- and short-term postoperative outcomes in patients, who underwent left-sided colectomies with either the Da Vinci Si and Xi robotic systems or a laparoscopic technique and to establish left-sided colectomies as a training procedure for low anterior resections for rectal cancer.

## Materials and methods

### Inclusion and exclusion criteria

The present study was approved by the local ethics committee [Ethikkommission Nordwest- und Zentralschweiz (EKNZ), Project-ID 2020-02969].

All patients aged ≥ 18 years requiring elective left-sided colectomy (left hemicolectomies, sigmoidectomies, or rectosigmoidectomies) with a minimally invasive approach [robotic-assisted laparoscopy (RAL) or laparoscopy (LSC)] between June 2015 and December 2019 were included in this study. Patients with benign diseases (e.g., chronic and recurrent diverticulitis, polyps that could not be resected endoscopically) as well as malignancies were included. Tumors graded T4 preoperatively were excluded because we prefer an open approach for large tumors with suspicion of organ infiltration. The retrospective analysis of prospectively collected data was performed at two high-volume, colorectal surgery units. RAL vs. LSC was chosen according to instrument availability and surgeons’ preference, with no specific selection criteria. All patients were preoperatively informed about the surgical technique.

### Data collection

Data were retrospectively obtained from written hospital records, electronic databases as well as pathology and radiology reports. Demographic data (age, sex, and BMI) as well as outcome parameters were extracted, including conversion to open surgery, intra- and postoperative complications (30-day-morbidity), operative time, diverting stoma formation, postoperative length of hospital stay (LOS), blood transfusions, postoperative length of intensive care unit (ICU) stay (LOI), re-operations, and discharge at home or to a rehabilitation center.

### Surgical technique

In RAL, a medial to lateral approach was most frequently performed (in selected cases lateral to medial as well). In the LSC resected patients, whether a medial to lateral or vice versa approach was used depended on the senior surgeon. In most patients, the splenic flexure was mobilized and, in nearly all patients, even for benign diseases an oncologic resection with central ligation of inferior mesenteric artery and vein was performed. Linear and circular stapling devices were routinely used to perform anastomoses via the double-stapling technique. The da Vinci Si® and Xi® robotic platforms (Intuitive Surgical, Sunnyvale, CA, USA) were used. Only one surgeon has had experience in robotic surgery when the robotic colorectal surgery program was started. All surgeons have been proctored repetitively by experienced international robotic colorectal surgeons.

### Statistical analysis

Descriptive statistics were presented as absolute frequencies for categorical variables and median with interquartile ranges (IQR) for continuous variables. The comparison of dichotomous values was performed with the chi-squared test, while continuous variables between groups were compared with the Mann–Whitney *U* test. Additionally, a propensity score matched (PSM) analysis with 1:2 ratio (RAL:LSC) was used to minimize the effect of confounders. Patients were matched according to age, sex, BMI, indication for surgery, and the da Vinci Xi® or Si® surgical system [18]. Two separate PSM analyses were performed for procedures during the learning curve or after having finished the learning curve. According to Parascandola et al., the learning curve for robotic- assisted, left-sided colectomies was estimated to be 35–45 operations [19]. Prolonged LOS was defined as longer than the overall mean value of 10 days. A *p* value < 0.05 was considered statistically significant. Statistical analysis was performed on MedCalc Statistical Software version 19.5.3 (MedCalc Software Ltd, Ostend, Belgium; https://www.medcalc.org; 2020).

## Results

### Patient characteristics

Between June 2015 and December 2019, a total of 683 patients were included. Patients underwent left-sided colonic resections with either RAL (*n* = 179, 26.2%) or LSC (*n* = 504, 73.8%). Mean age was 65.0 (56.0–72.0) years in the RAL group and 64.0 (55.0–73.0) years in the LSC group (*p* = 0.957). A total of 289 (42.3%) patients were male (RAL 40.1% vs. LSC 46.4%, *p* = 0.201). The mean BMI in the RAL group was 25.9 (23.3–28.7) kg/m^2^ vs. 25.2 (22.9–28.3) kg/m^2^ in the LSC group (*p* = 0.169). Indication for surgery was a malignant tumor in 23.6% (*n* = 161) of the patients (RAL 12.9% vs. LSC 27.4%, *p* < 0.001). The first PSM analysis served to identify homogeneous groups in terms of patients’ characteristics, and 528 patients were selected (176 in the RAL and 352 in the LSC groups). The second PSM analysis was performed to also take into account the type of robotic platform (da Vinci Xi® system) and the learning curve-related bias, resulting in 132 patients selected (44 in the RAL and 88 in the LSC groups). After propensity score matching, no difference in patients’ characteristics was noted between the groups. Details are displayed in Table 1.

**Table 1:** Patient characteristics

Variable	Overall analysis			PSM analysis			PSM analysis without learning curve operations		
	RAL (*n* = 179)	LSC (*n* = 504)	*p*	RAL (*n* = 176)	LSC (*n* = 352)	*p*	RAL (*n* = 44)	LSC (*n* = 88)	*p*
Age, years (IQR)	65.0 (56.0–72.0)	64.0 (55.0–73.0)	0.957	65.0 (56.0–72.0)	64.0 (55.0–72.0)	0.692	69.0 (62.0–73.0)	66.5 (56.5–74.5)	0.871
BMI, kg/m^2^ (IQR)	25.9 (23.3–28.7)	25.2 (22.9–28.3)	0.174	25.9 (23.3–28.7)	25.2 (22.9–28.5)	0.308	29.2 (27.6–33.2)	29.2 (27.6–33.2)	0.209
Gender, male (%)	83 (46.4)	206 (40.9)	0.201	82 (46.6)	152 (43.2)	0.458	26 (59.1)	54 (61.4)	0.802
Indication to surgery/malignant disease, n (%)	23 (12.9)	138 (27.4)	< 0.001	22 (12.5)	57 (16.2)	0.262	7 (15.9)	15 (17.0)	0.869

Values are expressed as absolute numbers with percentage in parentheses or median with interquartile range (IQR). *BMI* body mass index; *SD* standard deviation; *LSC* laparoscopic resection; *RAL* robotic-assisted laparoscopic resection; *PSM* propensity score matched

### Surgical outcomes

Intraoperative complication rate was not statistically different between the two groups (RAL 0.6% vs. LSC 2.0%, *p* = 0.193). Among intraoperative complications, four cases of bowel injury, three cases of bleeding (one in the RAL group), two lesions of the bladder, one ureter injury and a severe hypotension after pneumoperitoneum occurred. The conversion rate to open surgery was instead significantly higher in the LSC group (1.7% vs. 6.1%, *p* = 0.018). The 34 conversions were caused by adhesions in 19 cases, bowel injury in 4 cases, bladder injury in 2 cases, hypotension after pneumoperitoneum induction in 1 case, bulky tumor mass in 4 cases, technical problems in anastomosis formation in 1 case, hemorrhage in 1 case, ureter injury in 1 case, and obesity in 1 case. The PSM analyses confirmed the statistically significant difference between the groups. After classification according to Clavien–Dindo, most complications were Grade I or II, while only 7 (3.9%) patients in the RAL group and 32 (6.3%) patients in the LSC group suffered from complications graded Clavien–Dindo ≥ III (*p* = 0.227). Ileus was graded Clavien–Dindo II except for 1 patient who was readmitted at day 18 and needed revisional surgery for adhesive small bowel obstruction. Surgical site infections were graded Clavien–Dindo III when the wound was opened bedside under local anesthesia. Again, after PSM analyses, no statistically significant differences could be demonstrated. Details are reported in Table 2.

**Table 2:** Comparison of outcomes between patients undergoing RAL versus LSC

Variable	Overall analysis			PSM analysis			PSM analysis without learning curve operations		
	RAL (*n* = 179)	LSC (*n* = 504)	*p*	RAL (*n* = 176)	LSC (*n* = 352)	*p*	RAL (*n* = 44)	LSC (*n* = 88)	*p*
Intraoperative complications, *n* (%)	1 (0.6)	10 (2.0)	0.193	1 (0.6)	8 (2.3)	0.154	0	3 (3.4)	0.217
Operative time, min (IQR)	300 (250–323)	210 (180–260)	< 0.001	300 (250–325)	210 (180–260)	< 0.001	292 (258–323)	214 (180–256)	< 0.001
Conversion to open surgery, *n* (%)	3 (1.7)	31 (6.2)	0.018	3 (1.7)	20 (5.7)	0.035	0	7 (8.0)	0.050
Stoma formation, *n* (%)	1 (0.6)	5 (1.0)	0.594	1 (0.6)	2 (0.6)	1.000	0	0	1.000
ICU required, *n* (%)	46 (25.7)	81 (16.1)	0.004	46 (26.1)	47 (13.4)	< 0.001	15 (34.1)	19 (21.6)	0.123
ICU stay, days (IQR)	1 (1–2)	1 (1–2)	0.605	1 (1–2)	1 (1–2)	0.884	1 (1–3)	1 (1–2)	0.348
Blood transfusions, *n* (%)	5 (2.8)	11 (2.2)	0.215	5 (2.8)	6 (1.7)	0.389	2 (4.5)	0	0.045
Postoperative complications									
- Clavien–Dindo ≥ III, *n* (%)	7 (3.9)	32 (6.3)	0.227	7 (4.0)	23 (6.5)	0.232	1 (2.3)	5 (5.7)	0.377
- Anastomotic leakage, *n* (%)	2 (1.1)	8 (1.6)	0.653	2 (1.1)	5 (1.4)	0.788	0	3 (3.4)	0.217
- Length of hospital stay, days (IQR)	9 (8–11)	9 (7–11)	0.328	9 (8–11)	9 (7–11)	0.108	10 (8–11)	9 (8–11)	0.547
Reoperations, *n* (%)	4 (222)	16 (3.2)	0.522	4 (2.3)	11 (3.1)	0.579	0	3 (3.4)	0.217
Discharged home, *n* (%)	168 (93.8)	464 (92.1)	0.434	165 (93.7)	328 (93.2)	0.805	41 (93.2)	84 (95.5)	0.584

Values are expressed as absolute numbers with percentage in parentheses or median with interquartile range (IQR). *SD* standard deviation; *LSC* laparoscopic resection; *RAL* robotic-assisted laparoscopic resection; *ICU* intensive care unit

Of note, no difference concerning anastomotic leakage rate could be found between laparoscopic and robotic- assisted resections [RAL *n* = 2 (1.1%) vs. LSC *n* = 8 (1.6%), *p* = 0.653]. Reoperations were necessary in 2.4% of the patients after robotic-assisted surgery and in 2.2% of the patients after laparoscopic procedures (*p* = 0.522).

Operative time was significantly longer in the RAL group [300 (250–323) vs. 210 (180–260) minutes, *p* < 0.001], and this difference was also confirmed after PSM analyses. The frequency of stoma formation was similar in both groups (0.6% vs. 1.0%, *p* = 0.594). Details are reported in Table 3.

**Table 3:** Postoperative complications

Postoperative complications	RAL (*n* = 179)	LSC (*n* = 504)
Clavien–Dindo ≥ III		
·Anastomotic leakage, *n* (%)	2 (1.1)	8 (1.6)
·Surgical site infection superficial, *n* (%)	2 (1.1)	1 (0.2)
·Bowel perforation, *n* (%)	1 (0.6)	2 (0.4)
·Bile duct injury, *n* (%)	1 (0.6)	0
·Surgical site infection deep, *n* (%)	1 (0.6)	0
·Anastomotic bleeding, *n* (%)	0	7 (1.4)
·Cardiovascular, *n* (%)	0	3 (0.6)
·Ileus, *n* (%)	0	3 (0.6)
·Trocar site hernia, *n* (%)	0	3 (0.6)
·Pneumonia, *n* (%)	0	2 (0.4)
·Presence of foreign body	0	2 (0.4)
·Intraabdominal hematoma, *n* (%)	0	1 (0.2)

Values are expressed as absolute numbers with percentage in parentheses

The median length of ICU stay was similar in the RAL and LSC group [1 (1–2) vs. 1 (1–2) days, *p* = 0.605], but, interestingly, the proportion of patients requiring ICU admission was higher in the RAL group [46 (25.7%) vs. 81 (16.1%) patients in the RAL and LSC group, respectively, *p* = 0.004]. This difference was also confirmed in the first PSM analysis, including the learning curve. In the PSM analysis with exclusion of the learning curve procedures, no statistically significant difference could be demonstrated, probably because of the reduced sample size.

Length of hospital stay was similar in both groups [9 (8–11) vs. 9 (7–11) days in the RAL and LSC groups, respectively, *p* = 0.328]. Regardless of the type of surgery performed, most patients could be discharged home without the need for further rehabilitation or alternative health care facilities (93.8% vs. 92.1% in the RAL and LSC groups, respectively, *p* = 0.434).

To detect differences in outcome parameters for benign and malignant lesions, a third PSM analysis was performed. As expected, patients in the subgroup for malignancies were older than in the general analysis and male sex was more prevalent. Intra- and postoperative outcomes were similar to the overall analysis and no noteworthy differences could be found.

## Discussion

This analysis represents the data of two high-volume colorectal centers in Switzerland. A total of 683 patients (179 RAL and 504 LSC) were included, and data were analyzed for several outcome parameters.

Complications were classified according to Clavien–Dindo, complications graded higher or equal III were not statistically significantly different (7.7% RAL vs. 11.2% LSC, *p* = 0.160) in both cohorts. These results are comparable with a study from Dolejs et al. in 2017 [20]. The team reviewed a large, nationwide dataset of patients undergoing colectomies with primary anastomosis between 2012 and 2014 and sub-classified patients into different kinds of colorectal resections. Unfortunately, morbidity was not classified according to Clavien–Dindo but into two categories: “overall morbidity” and “serious morbidity”. Serious complication rate was 9% for patients undergoing left-sided laparoscopic resections compared to 7.2% for robotic-assisted resections, and no statistically significant difference could be demonstrated (*p* = 0.190). Strikingly, in our patient population, the proportion of anastomotic leakage and hemorrhage was higher in the laparoscopic group though not statistically significant. This remains unexplained since anastomotic technique and stapling devices were identical in both cohorts.

Concerning stoma formation one patient in the robotic-assisted group needed a stoma vs. five patients in the laparoscopic group (0.6% vs. 1.0%, *p* = 0.594), this difference failed to be statistically significant. Elliott et al. compared robotic-assisted vs. laparoscopic resections for sigmoid diverticulitis with fistula and found, in contradiction to our results, a higher rate of stoma formation in the RAL than in the LSC group [*n* = 2 (18%) vs. *n* = 0 (0%) *p* = 0.048] [21]. An explanation for the higher rate of stoma formation in the study by Elliott might be the newer technique of robotic-assisted resections and the wish to “protect” the surgical result or simply the small sample size and heterogenous patient cohort.

Conversion rate is a vital outcome parameter for surgical quality. After conversion to open surgery, patients have a longer hospital stay and an increased risk of developing postoperative complications as well as poorer oncological outcomes [22]. Furthermore, institutions and payers claim the additional costs of converted surgical procedures and longer hospital stay for the healthcare system [23]. Conversion rate in our analysis was significantly lower in in the RAL group compared to the LSC group (1.7% RAL vs. 6.1% LSC, *p* = 0.018). In 2016, Zhang published a meta-analysis comparing clinicopathological outcomes of robotic-assisted and laparoscopic colorectal surgery for cancer. Twenty-four studies with a total of 3318 patients were included and, in agreement with our data, a significantly lower conversion rate in the RAL group could be demonstrated [24]. The reason for the lower conversion rate in robotic-assisted resections might be the multiple technical advantages making it easier to cope with intraoperative difficulties like adhesions, narrow pelvis, or overweight patients. Furthermore, especially in the beginning of the era of robotic-assisted surgery, most surgeons performing robotic-assisted operations were experienced laparoscopic surgeons as well, which might have led to lower conversion rates, too. Finally, for a newly introduced technique, a selection bias for the straight-forward and easier cases should probably be considered as well.

Operative time was significantly longer for the RAL group compared to the LSC group. Several robotic-assisted surgery studies in various specialties confer to this result. In 2016, Ferrara et al. from Siena reported very similar operative times for a population of patients with any kind of colorectal surgery (RAL 293.6 vs. LSC 223.0 min) [25]. These findings are in accordance with a study by Raskin et al. from 2019. For patients with resections for diverticular disease operating room times were statistically significantly longer for the robotic-assisted group compared with the open group (256.5 ± 75.2 min. vs. 197.6 ± 74.3 min.) and for the robotic-assisted group compared with the laparoscopic group (254.4 ± 74.8 min. vs. 212.2 ± 75.0 min.) [26–29]. In our experience, a standardization of docking steps and repetitive training with identical teams are key points to reduce operative time. A confounding factor for the longer operative time might be the use of the da Vinci Si® platform for a relevant number of cases [*n* = 29 (16.2%) da Vinci Si® vs. *n* = 150 (83,8%) da Vinci Xi®]. This assumption is supported by a study from Hill and McCormick, which showed a significant reduction of operative times in, for example, sigmoidectomies when comparing the da Vinci Si® with the da Vinci Xi® platform (235 min. vs. 162 min., *p* = 0.0001) [30]. Surprisingly, in our study, the mean operative time for the Si® group was 290.96 min. (± 68.99), whereas the mean operative time for the da Vinci Xi® procedures was 296.91 min. (± 57.22). In the center with the da Vinci Xi® platform, five surgeons performed robotic-assisted operations, which means more patients were operated during the learning curve phase. For capacity reasons, after completing the learning curve, only demanding cases were scheduled for the robotic-assisted approach. This might explain the longer operative times for the da Vinci Xi® surgical system.

Our data analysis has shown a similar hospital stay for patients with robotic-assisted resections (10.3 days RAL vs. 9.9 days LSC, *p* = 0.434). This result is corroborated by a meta-analysis from Trin and coworkers. They analyzed and reviewed 14 studies on colorectal laparoscopic vs. robotic-assisted resections. In all studies, length of postoperative hospital stay was assessed; statistical analysis yielded no difference between the two approaches as well [11]. As a matter of fact, length of hospital stay is an increasingly important outcome parameter, since it has significant implications on procedure associated costs.

Over the last few years, more and more hospitals have started ERAS programs for colorectal surgery. In both colorectal centers ERAS programs have now been implemented. Nevertheless, data acquisition of the present study was completely finished before starting ERAS programs. This might explain the rather long hospital stay of our patients compared to current literature. The proportion of patients requiring ICU admission (comprising ICU, intermediate care unit, and recovery room) postoperatively was higher in the RAL group [46 (25.7%) vs. 81 (16.1%) patients in the RAL and LSC group, respectively, *p* = 0.004]. This result can be attributed to the initial policy of transferring every patient to the ICU for safety reasons in the beginning of the implementation of the robotic-assisted surgery program. In the further course, only complex resections which indeed needed ICU treatment were scheduled for robotic-assisted resections.

The present study has some limitations. The study character is retrospective and has no randomization. In a retrospective study design, data quality depends on correct acquisition and completeness of intraoperative details and documentation of the postoperative course. There may have been a selection bias between the RAL and the LSC groups, though multivariate analyses tend to compensate for this issue. Another confounder might be the learning curve and the unequal experience in minimally invasive techniques of the surgeons, which could contribute to longer operative times in the RAL group. Furthermore, the use of two different generations of robotic-assisted platforms in the two institutions might reduce comparability of the data as well. However, such potential biases related to selection of patients, type of da Vinci® robotic platform and the learning curve effect were addressed with a PSM analysis, which showed no noteworthy differences compared to the main analysis.

## Conclusion

Robotic-assisted laparoscopic resections for left-sided colectomies are safe and feasible and can therefore serve as training procedures for robotic-assisted low anterior resections. Intra- and postoperative complications are similar in both the RAL and LSC groups. Most notably, the rate of anastomotic leakages is similar. Compared to laparoscopic resections, the analyzed robotic-assisted resections have longer operative times but less need to conversion. Future prospective randomized trials with the latest da Vinci Xi® surgical system should be performed to mitigate confounders of the present study and to further establish the use of robotic platforms for left-sided colectomies.
